# A diagnosis model in nasopharyngeal carcinoma based on PET/MRI radiomics and semiquantitative parameters

**DOI:** 10.1186/s12880-022-00883-6

**Published:** 2022-08-29

**Authors:** Qi Feng, Jiangtao Liang, Luoyu Wang, Xiuhong Ge, Zhongxiang Ding, Haihong Wu

**Affiliations:** 1 Department of Radiology, Affiliated Hangzhou First People’s Hospital, Zhejiang University School of Medicine, Hangzhou, 310000 China; 2 Hangzhou Panoramic Medical Imaging Diagnostic Center, Hangzhou, 310000 China; 3 Translational Medicine Research Center, Key Laboratory of Clinical Cancer Pharmacology and Toxicology Research of Zhejiang Province, Affiliated Hangzhou First People’s Hospital, Zhejiang University School of Medicine, Hangzhou, 310000 China; 4 Chunan First People’s Hospital, Hangzhou, 310000 China

**Keywords:** Nasopharyngeal carcinoma, Positron emission tomography, Magnetic resonance imaging, Radiomics, Semiquantitative parameters

## Abstract

**Background:**

The staging of nasopharyngeal carcinoma (NPC) is of great value in treatment and prognosis. We explored whether a positron emission tomography/ magnetic resonance imaging (PET/MRI) based comprehensive model of radiomics features and semiquantitative parameters was useful for clinical evaluation of NPC staging.

**Materials and methods:**

A total of 100 NPC patients diagnosed with non-keratinized undifferentiated carcinoma were divided into early-stage group (I—II) and advanced-stage group (III—IV) and divided into the training set (n = 70) and the testing set (n = 30). Radiomics features (n = 396 × 2) of the primary site of NPC were extracted from MRI and PET images, respectively. Three major semiquantitative parameters of primary sites including maximum standardized uptake value (SUVmax), metabolic tumor volume (MTV), and total lesion glycolysis (TLG) in all NPC patients were measured. After feature selection, three diagnostic models including the radiomics model, the metabolic parameter model, and the combined model were established using logistic regression model. Finally, internal validation was performed, and a nomogram for NPC comprehensive diagnosis has been made.

**Results:**

The radiomics model and metabolic parameter model showed an area under the curve (AUC) of 0.83 and 0.80 in the testing set, respectively. The combined model based on radiomics and semiquantitative parameters showed an AUC of 0.90 in the testing set, with the best performance among the three models.

**Conclusion:**

The combined model based on PET/MRI radiomics and semiquantitative parameters is of great value in the evaluation of clinical stage (early-stage group and advanced-stage group) of NPC.

## Introduction

There were about 129,079 new cases of nasopharyngeal carcinoma (NPC) in 2018, most of which were concentrated in Southeast Asia and showed an increasing trend year by year [1]. In Asian countries, NPC patients with undifferentiated form of non-keratinized squamous carcinoma account for more than 95% in the endemic area [2]. The prognosis of NPC is poor due to the lack of understanding of its prominent symptoms. The treatment for NPC includes radiotherapy or chemotherapy or a combination of the two. For advanced and metastatic disease, surgical excision is usually the last option. Accurate staging of NPC is critical because it affects the patient's treatment, the plan of radiotherapy, and the prognosis.

The study of Orlhac et al. [3] showed that the tracer uptake values from positron emission tomography (PET) images can reflect the heterogeneity of tumors. PET‑computed tomography (PET/CT) using fluorine‑18 fludeoxyglucose (^18^F‑FDG) provides high spatial resolution and semiquantitative parameters. It is a widely used technique for NPC staging assessment and prediction of clinical outcomes [4–6]. Gu et al. [6] analyzed the clinicopathologic parameters and some PET parameters from pretreatment ^18^F‑FDG PET/CT images of 171 patients with NPC and constructed a prognostic nomogram. It has shown that intratumor heterogeneity can predict the long-term prognosis of patients with NPC. PET/magnetic resonance imaging (PET/MRI) has a high resolution of soft tissue, and has great advantages in the identification and delineation of tumor edges, as well as provides metabolic information. Some studies have shown the value of PET/MRI in tumor diagnosis and prediction. A study showed that ^18^F‑FDG PET/MRI may help to predict tumor grade and differentiate between types of intrahepatic neoplasms using apparent diffusion coefficient and standardized uptake value (SUV) [7]. Another PET/MRI study indicated that diagnostic model based on the combination of metabolic and texture parameters showed satisfactory performance with AUC of 0.961 in differentiating malignant from benign pancreatic cystic lesions [8]. It has been reported that PET/MRI has similar or even higher diagnostic accuracy than PET/CT in patients with NPC [9, 10]. However, there are relatively few PET/MRI studies on NPC.

Over the past decade, Artificial Intelligence (AI) algorithms have had a major impact on daily life. In addition to providing automated and standardized processes, AI can provide radiologists with new tools for quantitative analysis and image interpretation, which can save time and effort, improve diagnostic performance, and optimize the overall workflow. Radiomics is a rapidly developing new AI technique in medical imaging. A significant advantage of radiomics is the ability to obtain complete tissue information in a repeatable, non-invasive way. It can explore a tumor in its entirety (including its stromal microenvironment) and to perform longitudinal comparisons before and after one or more treatment rounds [11]. It can provide an economical and repeatable method for the study of tumor gene expression and metabolic state [12]. Radiomics has been widely used in cancer research, such as lung cancer [13], prostate cancer [14], glioblastoma [15], colorectal cancer [16], brain cancer [17], as well as NPC [18–20]. Yu et al. [18] conducted a MRI-based radiomics study on 70 patients with NPC (Stage II-IVB) using contrast-enhanced T1-weighted and T2-weighted MR images. This study successfully demonstrated the potential of MRI radiomics for pretreatment prediction of adaptive radiation therapy eligibility in patients with NPC. Zhao et al. [19] performed multiparametric MRI-based radiomics on 123 NPC patients and indicated that a radiomics nomogram combining the clinical data with radiomics signature can be used to predict the induction chemotherapy response before treatment in locally advanced NPC. However, there is still a lack of PET/MRI based radiomics studies on NPC staging. In this study, we will build and validate a combined diagnosis model based on ^18^F-FDG PET/MRI for NPC staging.

## Materials and methods

### Patients

The primary NPC patients whose pathologies were non-keratinized undifferentiated carcinoma examined in Hangzhou Universal Medical Imaging Diagnostic Center from June 2017 to October 2019 were collected. Prior to the PET/MRI examination, all patients signed informed consent, the study protocol met the requirements of medical ethics (No. KT2018024), and all methods were implemented in accordance with the Declaration of Helsinki.

Inclusion criteria were as follows: (1) The nasopharyngeal lesion was found for the first time, and no anti-tumor therapy such as chemotherapy or radiotherapy was performed; (2) NPC patients with non-keratinized undifferentiated carcinoma confirmed by pathology; (3) Able to cooperate well with whole body and head and neck PET/MRI examination. Exclusion criteria were as follows: (1) Suspected deviation of SUV value (e.g., hyperglycaemia, low radiation purity of FDG drug); (2) Patients with other systemic malignancies; (3) PET or MRI images do not meet diagnostic criteria; (4) Patients who had received any form of treatment prior to the PET/MRI examination.

Finally, a total of 100 primary NPC patients were collected according to the inclusion and exclusion criteria. These patients were divided into early-stage group (I—II) and advanced-stage group (III—IV) according to the 8th edition of the American Joint Committee on Cancer (AJCC)/Union for International Cancer Control (UICC) TNM system [21].

### ^18^F–FDG PET/MRI Examination

Image data were collected using GE integrated time-of-flight (TOF)-PET/MRI (GE SIGNA, Wisconsin, USA). The system consists of a PET detector with TOF technology and the latest generation of 750 W 3.0 T MR. The TOF-PET detector consists of a state-of-the-art solid-phase array photoconverter and a new generation of lutetium based scintillator (LBS) crystals. Horizontal field of view (FOV): 60 cm, axial FOV: 25 cm, horizontal resolution: 4.2 mm, axial resolution: 5.8 mm, time resolution: 385 ps, energy resolution: 11%, sensitivity: 21 cps/kBq, the thinest acquisition layer thickness: 2.8 mm, to achieve PET and MR synchronous scanning. The developer was ^18^F-FDG, with radiochemical purity ≥ 95%. Patients were deprived of food for more than 6 h, strenuous exercise was prohibited before injection of ^18^F-FDG, peripheral blood was collected to measure blood glucose concentration, and blood glucose was controlled below 7.8 mmol/L. The patient was injected with ^18^F-FDG at a dose of 3.7Mbq /kg, rested in the darkroom for 40 min, and underwent PET/MRI examination after urination. Whole-body PET/MRI scan was performed from the top of the head to the middle part of the femur. PET performed 3D mode, TOF and point spread function for acquisition and reconstruction. Subsequently, a local PET/MRI scan of the head and neck was performed from the skull base to the two clavicles. Special MRI coils were used in the head and neck areas to obtain cross-sectional, coronal and sagittal images. The scan parameters for PET images were as follows: using the Ordered Subset Expectation Maximization (OSEM) with 5 iterations with a 192 × 192 image matrix and a voxel size of 1.5625 mm × 1.5625 mm × 2.78 mm. The scan parameters for cross-sectional T2 iterative Dixon water-fat separation with echo asymmetry and least-squares estimation (IDEAL) sequence were as follows: image matrix = 256 × 256, a voxel size = 0.93 mm × 0.93 mm × 4 mm, repetition time (TR) = 4800 ms, echo time (TE) = 88 ms, slice thickness (ST) = 4 mm, FOV = 220 × 220 mm2, scanning time = 03:37 min. All data were collected from the same PET/MRI scanner. In this study, local cross-sectional T2 weighted image (T2WI) and PET images of head and neck were selected for later analysis.

### Semiquantitative parameters measurement

Each metabolic parameter was measured using PET VCAR software in GE Healthcare AW 4.6 post-processing workstation by a neuroradiologist with 12 years of work experience. Image analysis includes visual analysis method and semi-quantitative analysis method. The PET/MRI images of local head and neck scanning were opened, the PET/MRI fusion images, the cross-sectional, coronal and sagittal PET images were opened in a 4 × 4 window, respectively. We used the adaptive threshold method [22] to determine 40% of maximum SUV (SUVmax) in ROI as the tumor boundary. The ROI automatic identification box was inserted and placed in the outline of the nasopharyngeal lesion, and the tumor boundary was drawn through the iterative adaptive algorithm. The size of the identification box was adjusted in different windows of three directions, and the normal tissue and high uptake areas such as metastatic lymph nodes were excluded from the ROI range. Finally, three semiquantitative parameters of ROI including SUVmax, metabolic tumor volume (MTV), and total lesion glycolysis (TLG) are generated automatically by the software.

### Radiomics signature building

Image preprocessing was conducted using Artificial Intelligence Kit software that complied with imaging biomarker standardization initiative (IBSI) guidelines which were described at our previous study [23]. The spatial resampling was adjusted to 1 mm × 1 mm × 1 mm. The image was transformed into 1 mm layer thickness. Then, image gray was adjusted to 0–255. ITK-SNAP software (http://www.itksnap.org/pmwiki) was used for 3D region of interest (ROI) segmentation. We fused PET images into T2WI images to outline ROI. The primary NPC lesions were manually segmented layer by layer on the T2WI image by a neuroradiologist with 12 years of work experience. The segmentation boundaries of the PET images and the T2WI images were coincident. The details of the ROI segmentation were described at our previous study [23].

Import all preprocessed data and ROI data of T2WI and PET into Artificial Intelligence Kit software respectively. The radiomics parameters to be calculated include Histogram, Formfactor, Gray level cooccurrence matrix (GLCM), Haralick, and run-length matrix (RLM). The checked step sizes for GLCM and RLM are 1, 4, and 7. Our previous study showed the meaning of the calculated parameters [23]. Then, the 100 NPC patients were randomly allocated to the training set (n = 70) and testing set (n = 30).

The T2WI and PET data were combined for feature selection with 792 features. Two feature selection methods were used: maximum relevance and minimum redundancy (mRMR) and least absolute shrinkage and selection operator (LASSO). The principle of mRMR is maximize the correlation between features and categorical variables and minimize the correlation between features and features. First, we used mRMR to eliminate redundant and irrelevant features. The LASSO regression model was then used to reduce the dimension and the tenfold cross validation of the minimum standard was used.

### Development and validation of the radiomics model

Three diagnostic models were developed using logistic regression model: (1) the radiomics model was build using above selected radiomics features; (2) the metabolic parameter model was build using the three semiquantitative parameters (SUVmax, MTV, and TLG); (3) the combined model was performed combining the selected radiomics features and two of the three semiquantitative parameters. Internal validation was performed, and the performance of the three models was assessed using the ROC curves. Here, we performed DeLong’s test using MedCalc software to compare whether the ROC curves were different. Subsequently, a nomogram for NPC staging (early-stage group (I—II) and advanced-stage group (III—IV)) diagnosis has been made. The calibration curves were performed describing the alignment between the predicted probability of advanced NPC and the observed advanced results. Finally, we used decision curve to evaluate the clinical usefulness of the model.

### Statistical analysis

For analysis of clinical data comparison, either Student’s *t*-test or Mann Whitney’s *U* test was performed for continuous variables. Chi-square test was used for comparison of counting data. These above statistical analyses were performed using SPSS (version 22.0, IBM). All statistical analyses about radiomics analysis and model building were performed using the Artificial Intelligence Kit software and the R software (version 3.5.2; http://www.Rproject.org).

## Results

### Patient characteristics

Patient characteristics in the training group (n = 70) and the testing group (n = 30) were given in Table 1. There were no significant differences between the two groups in age, gender, the three semiquantitative parameters (SUVmax, MTV, TLG), and the clinical stage with *P* > 0.05.

**Table 1:** Patient characteristics in the training group and the testing group

	Training group	Testing group	Statistics	*p* value
Age, mean ± SD, years	52.23 ± 12.33	50.40 ± 13.68	-0.658	0.512
Gender, Male: Female	56: 14	22: 8	0.544*	0.461*
SUVmax	10.91 ± 4.76	10.03 ± 3.78	-0.899	0.371
MTV	9.87 ± 7.38	11.31 ± 9.29	0.824	0.412
TLG	51.06 ± 46.95	57.61 ± 41.46	0.661	0.510
Clinical staging, I: II: III: IV	5: 14: 38: 13	2: 6: 19: 3	1.250*	0.767*

The measurement data were expressed as mean ± standard deviation (SD). Statistical methods: *t*-test and chi-square test (*)

### Radiomics signature

**Fig. 1:**
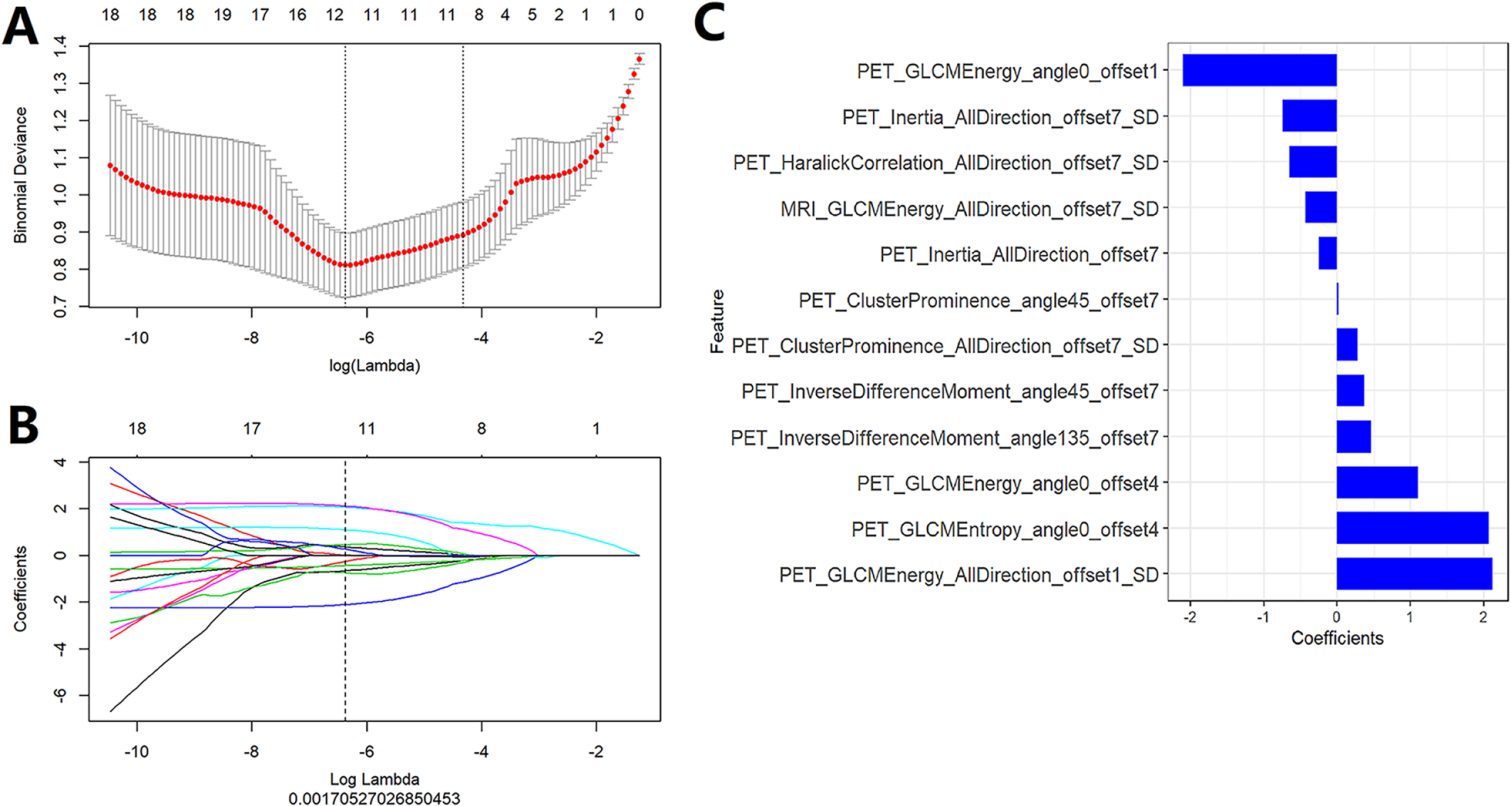
**A** and **B** are of the LASSO process. The vertical line is drawn at the optimal λ value using the minimum criterion and the standard error of the minimum criterion. Put the optimal λ value into **B** to get the best parameters. **C** Shows the remaining radiomics features after two steps of feature selection

We extracted 396 radiomics features from the T2WI images and PET images respectively, and a total of 792 features were obtained after integration. After mRMR feature selection, there were 20 radiomics features remained. Finally, after LASSO dimension reduction, there were 12 features remained (1 of T2WI images and 11 of PET images), as shown in Fig. 1.

### Classification performance

**Fig. 2:**
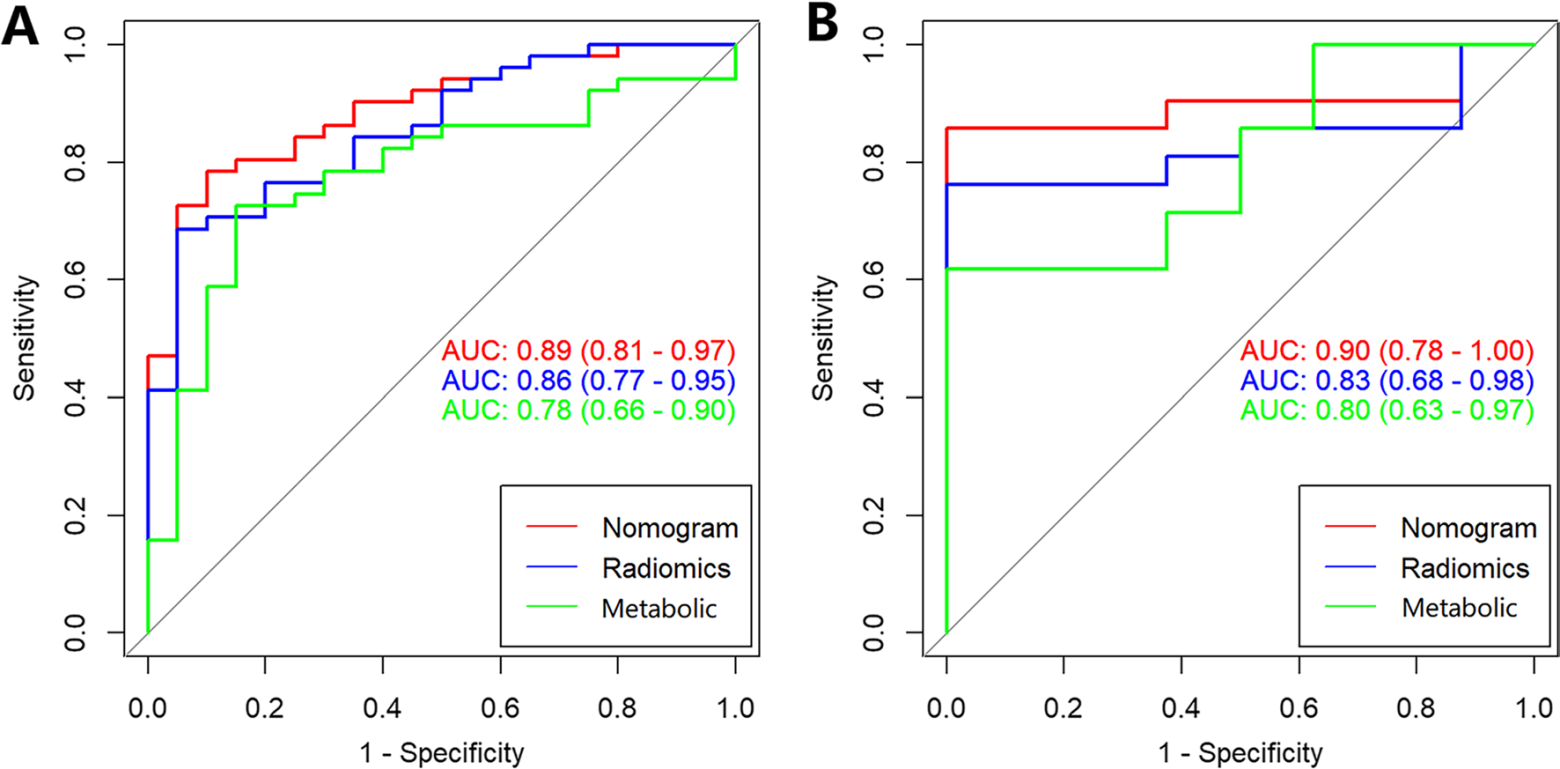
ROC curves of the radiomics model (blue line), metabolic parameter model (green line), and the combined model (red line). **A** ROC curves of training cohort. **B** ROC curves of testing cohort

**Fig. 3:**
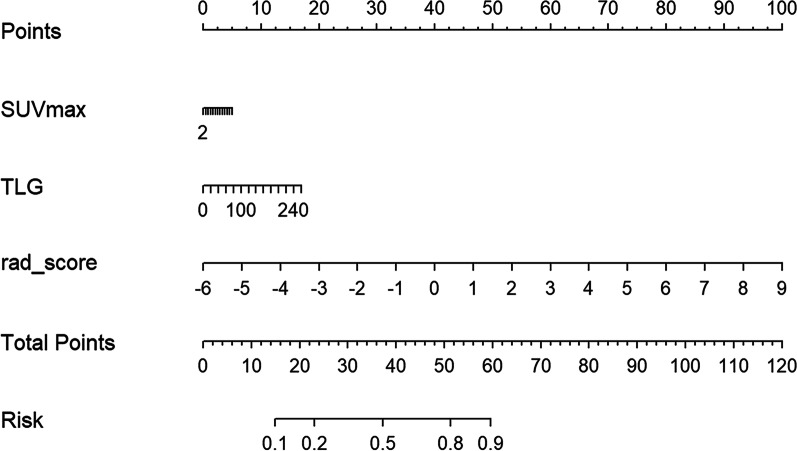
Nomogram for predicting NPC staging. For each patient, the values of the two semiquantitative parameters (SUVmax and TLG) and the radscore are evaluated by projecting them onto the topmost point scale. Add up the three variables and project the total score down to the bottom total points line to determine the risk of advanced NPC probabilities

**Fig. 4:**
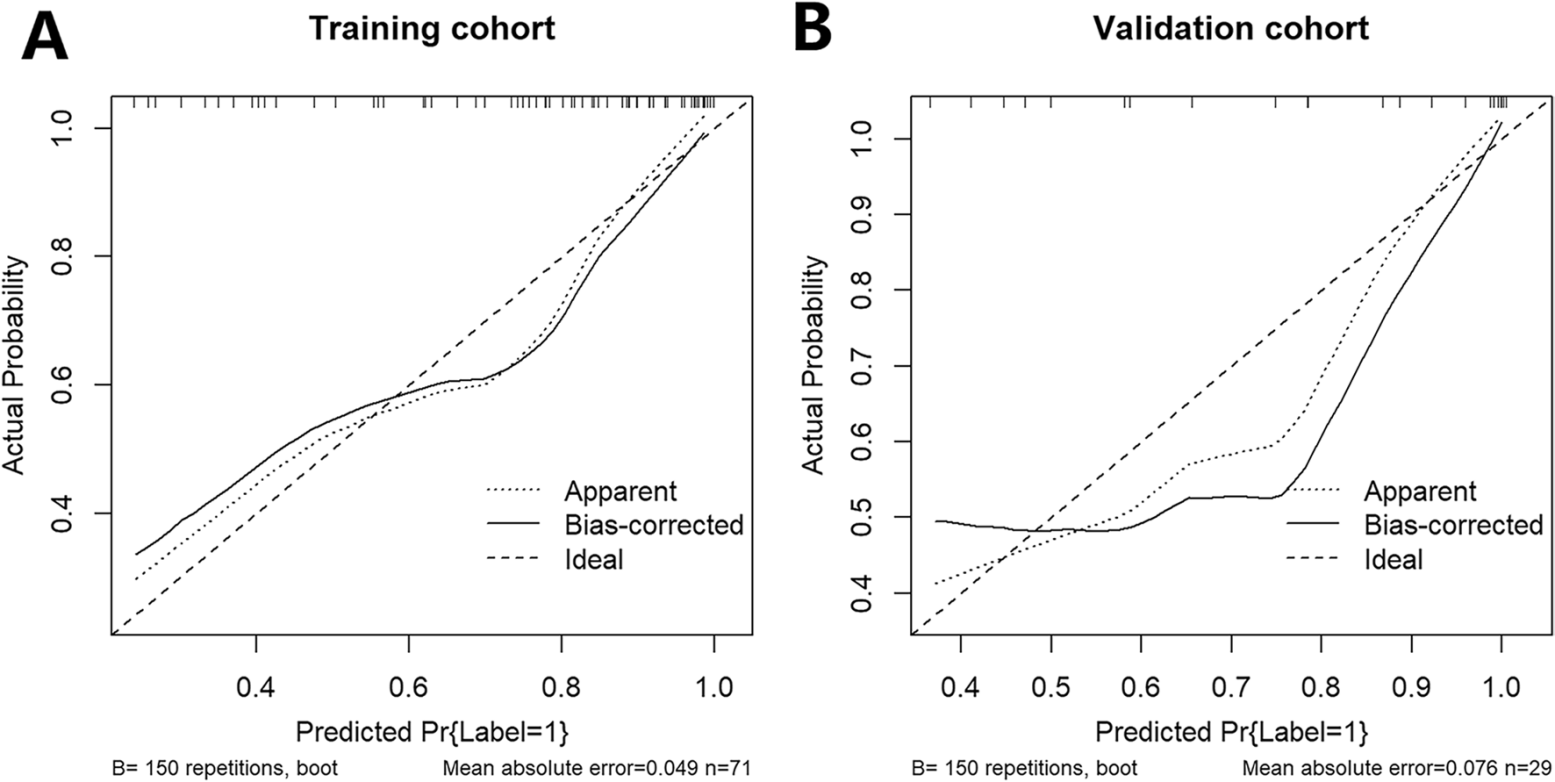
Calibration curve of the radiomics nomogram in training data (**A**) and testing data (**B**). The calibration curve describes the alignment between the model predicted probability of advanced NPC and the observed advanced results. The Y-axis represents the actual rate of advanced NPC. The X-axis is the advanced risk of the prediction. The dotted line on the diagonal represents the precise prediction of the ideal model. The other dotted line represents the apparent performance of nomogram, and the solid line represents the bias-corrected performance of nomogram. The higher the fit between the solid line and the diagonal dotted line, the better the prediction

The radiomics model and metabolic parameter model showed an area under the ROC curve (AUC) of 0.83 and 0.80 in the testing set respectively. The combined model based on radiomics and semiquantitative parameters showed an AUC of 0.90 in the testing set. Figure 2 showed the ROC curves of the three models. DeLong’s test showed that the ROC curves were statistically different between the combined model and clinical model with *P* value < 0.05. Based on Youden Index, other parameters of training and testing groups were showed in Table 2**,** including accuracy, sensitivity, specificity, positive predictive value, and negative predictive value. The cutoff of radiomics model, semiquantitative parameters model, and nomogram model were 1.13, 0.82, and 1.39, respectively. The combined model was presented as the nomogram (Fig. 3). The calibration curves of the radiomics nomogram in the training and testing cohort were showed in Fig. 4.

**Table 2:** Other evaluation parameters of training and testing groups in the three models

	Radiomics		Semiquantitative parameters		Nomogram	
	Training	Testing	Training	Testing	Training	Testing
Accuracy	0.76	0.83	0.76	0.66	0.82	0.90
Sensitivity	0.69	0.76	0.73	0.52	0.78	1.00
Specificity	0.95	1.00	0.85	1.00	0.90	0.73
Positive predictive value	0.97	1.00	0.93	1.00	0.95	0.86
Negative predictive value	0.54	0.62	0.55	0.44	0.62	1.00

### Clinical use

**Fig. 5:**
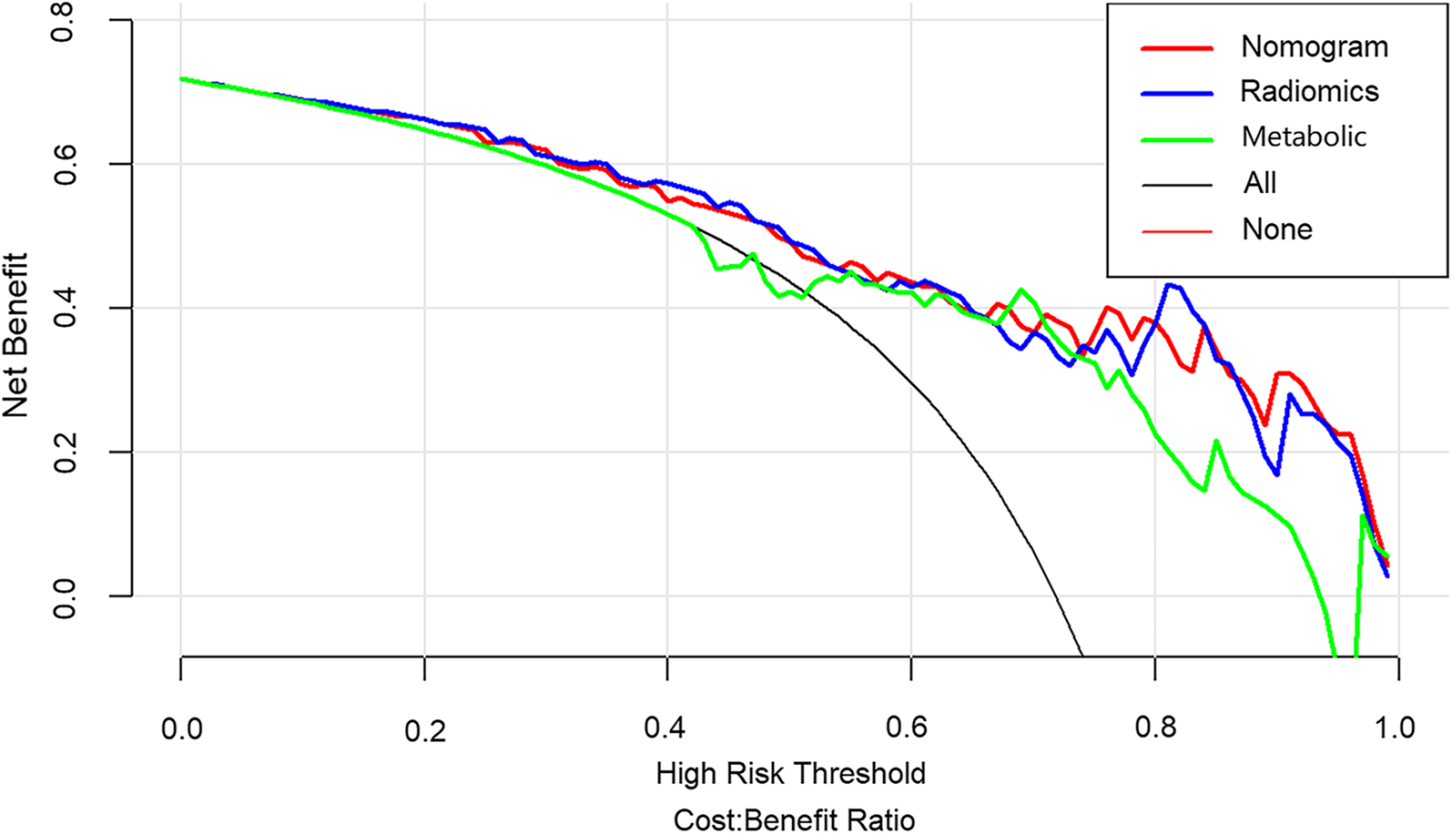
The decision curves containing nomogram, radiomics, and semiquantitative parameters. The decision curve showed that if the high risk threshold of a patient or doctor is > 15%, using the nomogram to predict advanced NPC adds more benefit than the treat-all-patients or the treat-none

Finally, we used decision curve to evaluate the clinical usefulness of the model (Fig. 5). The decision curve showed that if the high risk threshold of a patient or doctor is > 15%, using the nomogram to predict advanced NPC adds more benefit than the treat-all-patients or the treat-none.

## Discussion

Microstructural changes of primary NPC and clinical staging based on PET/MRI have not been fully studied. We extracted 12 radiomics features most relevant to clinical staging from T2WI and PET images of primary NPC, and discussed the value of radiomics features based on ^18^F-FDG PET/MRI combined with semiquantitative parameters in the evaluation of NPC staging. The results showed that the nomogram based on PET/MR had a high diagnostic efficiency in the staging of NPC.

After feature selection, the 12 remained features include 1 MR feature and 11 PET features. T2WI images may reflect tumor heterogeneity more objectively under the background of high signal of water molecules, and the selected characteristic parameters may be more significant. The feature of T2WI images belongs to GLCM parameter. GLCM represents the image texture formed by the repeated occurrence of voxel gray levels at spatial positions, and describes the joint distribution of two voxels gray levels with a certain spatial position relationship. The 11 features of PET images contain GLCM parameters, Texture parameters, and Haralick parameters. Texture is an important feature for identifying ROI images. Texture represents the appearance of a surface (e.g., smooth, rough…, etc.) and how its elements are distributed. Haralick is a statistic calculated based on GLCM. It is the sum of mean values calculated by selecting GLCM from four different directions. In addition, the semiquantitative parameters that ultimately entered the nomogram in our study were SUVmax and TLG. The most important semiquantitative parameters in ^18^F-FDG PET are SUVmax, MTV, and TLG, which can reflect the local invasion of tumor and the risk of distant metastasis. Studies have shown that SUVmax can also act as an independent prognostic factor for patients with NPC [24].

Radiomics is the study of high-throughput extraction and quantitative features analysis of medical images that are typically not available through visual evaluation. Data such as standard CT and MRI, as well as data from more complex cross-sectional imaging, can be combined to construct specific radiomics features, improving patient stratification and helping to personalize treatment [25]. Radiomics studies have demonstrated the relationship between quantitative image characteristics and gene expression patterns in cancer patients for the first time [26], thus assisting the diagnosis, treatment and prognosis assessment of cancer. Radiomics was already used for NPC diagnosis and prediction in previous studies. Yang et al. [27] studied the axial contrast-enhanced T1WI and T2WI MRI of 224 patients with locoregionally advanced NPC, and the multidimensional nomogram combining TNM stage, dose volume histogram parameters and radiomics showed high performance for predicting progression-free survival. Zhang et al. [28] performed radiomics nomogram incorporating MRI radiomics features and the TNM staging system, indicating that the nomogram improved prognostic ability in advanced NPC over the TNM staging system. A recent study analyzed pretreatment ^18^F-FDG PET/CT texture parameters of 52 patients with metastatic NPC, showing that it can provides complementary information to EBV DNA titers [4]. However, very few of these NPC studies involve PET/MRI data. A recent study involving 21 NPC patients analyzed robust features extracted from tumor volumes on PET/MRI images, indicating that it can provide guidance for building reproducible radiomics models for future NPC studies [20]. However, no further modeling was performed in this study. We build a comprehensive model that integrated PET/MRI radiomics and PET semiquantitative parameters based on the previous study [23]. Meanwhile, our model showed good performance not only in the training cohort, but also in the testing cohort.

There were still several limitations in our study. Firstly, the sample size of this study is relatively small, and the data comes from a single center. In the future, large samples and multi-center trials are needed for verification. Secondly, In ROI segmentation, we fused PET images into T2WI images to outline ROI. However, MRI volumes often does not match with metabolic volumes [29]. And we still choose manual ROI segmentation method which may carries a certain amount of subjectivity. Finally, this study only focused on the staging of NPC. We will further study the therapeutic efficacy monitoring, prognosis prediction, and correlation between radiomics features and genes based on the large sample PET/MR data.

In conclusion, the developed model combining radiomics based on ^18^F-FDG PET/MRI and PET semiquantitative parameters was valuable for NPC staging. With model improvement and external validation, it can be further supplemented for more accurate clinical NPC staging. In the future, radiomics may become a more useful and economical tool to predict NPC invasiveness, distant metastasis and therapeutic effect.

## Data Availability

The datasets generated and analyzed during the current study are not publicly available due to temporary inconvenient but are available from the corresponding author on reasonable request.

## Abbreviations

NPC: Nasopharyngeal carcinoma;
PET/CT: Positron emission tomography‑computed tomography
^18^F‑FDG: Fluorine‑18 fludeoxyglucose
PET/MRI: Positron emission tomography/magnetic resonance imaging
SUVmax: Standardized uptake value
MTV: Metabolic tumor volume
TLG: Total lesion glycolysis
ROI: Region of interest
GLCM: Gray level cooccurrence matrix
RLM: Run-length matrix
mRMR: Maximum relevance and minimum redundancy
LASSO: Least absolute shrinkage and selection operator
